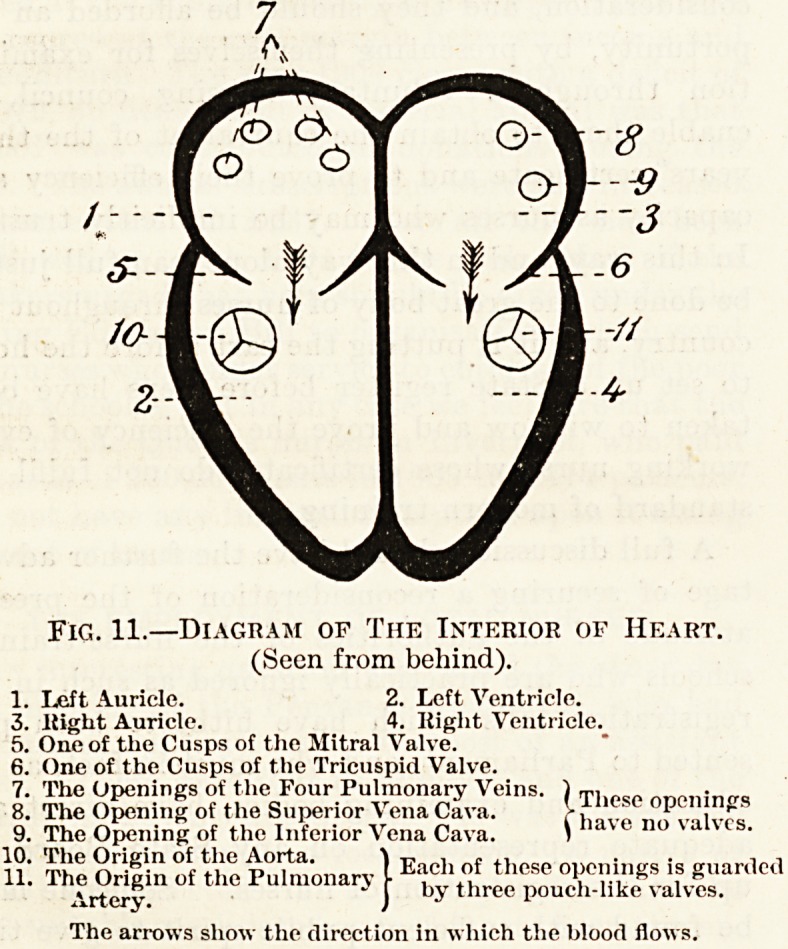# The Hospital. Nursing Section

**Published:** 1906-03-24

**Authors:** 


### The Hospital.

### Nursing Section.

Contributions for "THE HOSPITAL," should be addressed to the EDITOR, "THE HOSPITAL" NURSING SECTION, 28 & 29 Southampton Street, Strand, London, W.C.

No. 1,017.—VOL. XXXIX. SATURDAY, MARCH 24, 1906.

### Notes on News from the Nursing World.

### GUY'S HOSPITAL NURSES' LEAGUE.

THE annual meeting of the Guy's Hospital Nurses' League was held on Tuesday evening in the Nurses' Home. Many of the past members were present, also Sir Cooper and Lady Perry, and a large number of the sisters and nurses. Tea and coffee were served at 7.30 P.M., and the meeting commenced at 8.15. The minutes of the last annual meeting having been read, the matron, Miss Swift, said a few words of encouragement and advice. This was followed by one or two other speeches, and finally the result of the election of various sisters and nurses as representatives of the different sections of the Recreation Society was made known. These include photography, swimming, tennis, cycling, debating, and also a musical and literary section. Votes of thanks to the matron, the secretary, and retiring members were passed, and the meeting closed with a set of views thrown on the screen representing hospital scenes and various outside subjects of sea and land. The most prominent feature of the evening was the exhibition of the photographs taken by the nurses and one or two others connected with the hospital during the past year. Mr. Bailey, editor of *Photography*, kindly judged the photographs, and prizes were given for each class, consisting of handsome bronze plaques framed in brown oak. This branch of the League has flourished, and Mr. Bailey remarked that the work was excellent, especially considering that it was done by those whose time and energies are so fully occupied. The photograph gaining first prize was that of a hospital taken last summer.

### MATRON'S COURSE IN AUSTRALIA.

A VERY encouraging interest has been manifested in the matrons' course in Melbourne, and at the first lesson in cookery given under State direction, in February, the different branches of the nursing profession were well represented. There were two hospital matrons, the lady superintendent of the Australian Army Nursing Service, and an Army sister, the sister-in-charge of the District Nursing Society, a surgical sister, a sister from a private lunatic asylum, a sister from the Infectious Diseases Hospital, one probationer, and two private nurses. Two State school teachers completed the list. The syllabus for the first term, extending over ten weeks, is: (1) Roasting; (2) Vegetables; (3) Soups; (4) Pastry; (5) Fish, and wet and dry frying; (6) Boiling and Stewing; (7) Puddings; (8) Cakes; (9) Breakfast and tea; (10) Invalid. The kitchen arrangements were perfect, and though, with the thermometer at 96° in the shade, it was difficult to get up any enthusiasm on such a subject as roasting, with demonstrations, everyone felt that a new era in usefulness had begun.

### IRISH NURSES AND THEIR GRIEVANCES.

A FORTNIGHT ago we intimated our inability to gather, from the letters contributed to the Irish daily Press, the nature of the alterations desired by Irish nurses complaining of grievances, and laid stress upon the failure of the correspondents to cite specific cases of hardships. One of these correspondents, who confesses that she is the author of two letters, the first arraigning the matron of an isolation hospital and the second complaining of the work and food in a private hospital, informs us that she recited her actual experiences, but proceeds to state that it is between five and six years since she spent six months at a sanatorium in the east of England, and about four years since she was employed in a large private hospital in Dublin. We do not impugn her accuracy, but we do not think that it is fair to existing authorities of institutions to tender experiences which are wholly out of date as if they were evidence of what is necessarily going on to-day.

### THE HOLIDAY QUESTION.

THE Brighton Guardians have had before them the question of holidays for charge nurses. Hitherto, the annual leave of absence has been fourteen days, and the Management Committee which inquired into the matter, reported in favour of making it seventeen days. But several Guardians, evidently agreeing that it is not desirable to take two bites at a cherry, urged that the leave should be forthwith extended to twenty-one days, and in the end they carried their point by 16 votes to 12. An opponent of the extension observed that "many Brighton ratepayers would be pleased to have seventeen days' holiday per annum, with the knowledge that their income would continue unaltered." We are at a loss to understand in what way this bears upon the question. If the charge nurses at the Brighton Poor-law Infirmary need twenty-one days' holiday, we do not believe for a moment that the vast majority of the ratepayers will grudge it to them.

### THE EXETER GUARDIANS AND THEIR SUPERINTENDENT NURSE.

WE observe from a local paper that one of the Exeter Guardians has been condemning what he is pleased to term "an inspired paragraph" in our columns of March 10, to the effect that the superintendent nurse at the Workhouse Infirmary is entirely under the direction of the medical officer in regard to interfering with the thermometers, or the quantity of fuel used. Some of his colleagues having deprecated notice being taken of "newspaper paragraphs," he justified his action by the remark that in the absence of any explanation, the Guardians might be regarded as a set of busybodies, and affirmed that the reduction of the fuel consumed during the past month abundantly warranted the course which had been pursued. We regret, however, that the Guardians have endorsed a report of the Management Committee which is virtually an incitement to the workhouse master to continue to interfere in matters which ought to be outside his jurisdiction, and we shall be surprised if the medical officer and the superintendent nurse do not insist that the wards shall be entirely under their control.

### ST. JOHN'S NURSES AND THE TRAINING OF MIDWIVES.

In the report for 1905 of the Community of the Nursing Sisters of St. John the Divine, the Superior dwells upon the necessity of an advance in the standard of training for midwives. Pointing out that St. John's nurses are not allowed to enter upon that kind of training until their three years' general work is completed, the Superior says that usually this is not the case, and she sees numbers of women passed who have not necessarily had any nurse training at all—or at most very little. She correctly asserts that other countries, far poorer, less advanced in other ways, and far less able to bear the expense, will not tolerate this, and insist upon their midwives being properly trained. Certainly, the fact that the St. John's nurses, all fully trained, attended 900 maternity cases last year in the district homes without one death among the mothers, is a most powerful argument in favour of midwives being also trained nurses.

### THE DANGERS OF A LITTLE KNOWLEDGE.

In the colony of Newfoundland a few weeks ago a lecture was given to a local branch of the Girls' Friendly Society in Home Nursing. The lecturer dwelt strongly on the importance of ventilation and fresh air, her remarks having reference to an epidemic of measles from which the town was suffering at the time she was speaking. Her insistence on the evil of crowding four or five children together in a small room without any ventilation evidently created a considerable impression. It also provoked immediate action, one of the members of the Society returning to her home where a child was ill with measles, observing that "Miss —— said that people with measles should have lots of fresh air," opened a window close to the patient. The weather was very cold, the thermometer far below freezing, and the child developed pneumonia. When the medical man attending the case learnt what had happened, he did not bestow his blessing upon the lecturer on "Home Nursing."

### THE NORTH LONDON NURSING ASSOCIATION.

With reference to our remarks last week respecting the annual meeting of the North London Nursing Association, the Chairman and the Hon. Secretary sends us a courteous letter which we insert with pleasure. We are glad to learn from him that the present unfortunate financial position is considered only temporary, and we note with satisfaction that in addition to the ball on behalf of the organisation, a sale of work by the nurses is being arranged. These indicate that vigorous efforts will be made this year to put the association in a sound financial position. As to the excellence of the work done by the nurses, with a capable superintendent at their head, there is no question; and we observe with interest that members of the staff who suffered through overwork from time to time derived great benefit from nursing for a few weeks at Horsted Keynes. The Committee earnestly appeal for more annual subscribers, and in this direction there is clearly room for improvement.

### MIDWIFERY IN CHINA.

The matron of Shantung Road Hospital, Shanghai, who went to the Far East from the Women's Hospital at Melbourne, relating some of her experiences of midwifery in China, says: "No man attends a Chinese woman, only a midwife, compared with whom the most atrocious of our home Gamps is an angel of cleanliness and skill and common sense. This atrocity in human form guarantees to bring the patient safely through, otherwise she gets no pay. Frequently, when the patient has undergone ill-treatment at her hands for several days, and is just about at the last gasp, someone suggests "the foreign hospital"; thus we get cases such as home nurses can have no conception of. One case, I remember, the midwife and patient came along, the patient on a door, the midwife walking beside, carrying in a piece of paper an arm, which had evidently been born, and which she had cut off because she had no idea what to do with it, and, as she expressed it, 'Could not make the baby come with that arm in the road.'"

### PNEUMONIA PATIENTS AND DELIRIUM.

An inquest was held at Stepney on Saturday respecting the death of a young man of twenty-seven, who had been a patient in the Poplar and Stepney Sick Asylum. According to the evidence of one of the sisters he became very feverish and queer in the head. His bed was against the eight-feet partition, and during her absence from the ward the deceased climbed over it and fell into the next ward on to his head. The evidence of the assistant medical officer was to the effect that the patient was slightly delirious owing to an attack of acute pneumonia, and that death was due to syncope and shock from the fall from the partition. We do not for a moment suggest any neglect on the part of the sister, but it is never safe to leave pneumonia patients who are suffering from delirium.

### AN UNWISE MARRIAGE.

The case of a man who was charged at Dewsbury last week with bigamy is interesting because the second wife he married was a nurse in Dewsbury Workhouse Infirmary. He was an inmate of that institution, and it appears that a friendship was struck up between them, which resulted in a proposal of marriage. Nurses sometimes marry their patients and do not regret their action, but in this instance the nurse had to lend the ex-patient money for a special license and also for the wedding ring. It is amazing that in these circumstances she should have consented to marry him. A man who has not even enough money to pay for the wedding ring must be most undesirable as a husband, though there may be no reason to suspect that he is capable of crime.

### QUEEN'S NURSES IN LIVERPOOL.

We cannot believe that the people of Liverpool will allow the Queen Victoria Nursing Association in that city to continue in its present unsatisfactory financial position. If only out of respect for the memory of William Rathbone, the generous pioneer of the movement, they will make haste to turn a deficit of £194 into a surplus. Moreover, as it was stated at the annual meeting in Liverpool, this does not represent the real margin between income and expenditure. The year 1904 closed with a deficit of £2,079, an dthe result of a special appeal was that £2,681 was contributed in donations during the year. The annual subscriptions were also increased to the extent of £237. Yet all this has been swallowed up, with £194 to the bad. Most of the speakers urged that help should be given under the coming Education Bill to organisations which send out nurses who render service to children of the poor in the schools; but in any case we feel sure that the work of the Queen's nurses in Liverpool, who paid upwards of 200,000 visits in 1905 to 8,414 patients, will not have any limitation imposed upon it owing to want of means.

### THE EMPLOYMENT OF LOCAL NURSES.

An interesting question was raised the other day at a meeting of the Penzance Guardians, who had to consider applications for the post of an assistant nurse. These were four in number, and one of the Guardians having urged that "it was always the best course to employ a local person," a native of Gulval was selected. It is quite possible that the most suitable candidate has in this case been chosen. But we are by no means in agreement with the view that local nurses should always have the preference. On the contrary, there are times when it is most desirable that new blood should be introduced into an institution, and it then becomes the duty of the authorities to select someone who is acquainted with methods obtaining in other parts of the country. This does not, of course, apply so much to assistant nurses in Poor-law infirmaries or sick wards as to sisters and matrons, but it may be worth while to urge that pursuance of the policy laid down at Penzance as a general rule would inevitably tend to retrogression.

### FOR THE QUEEN'S JUBILEE INSTITUTE.

On Tuesday evening, April 3, an amateur performance of "Billy's Little Love Affair," by H. V. Esmond, will be given at the Apollo Theatre on behalf of Queen Victoria's Jubilee Institute for Nurses. This is one of the special efforts of the ladies constituting Queen Alexandra's Committee, and we hope that the attendance will be large enough to secure a very substantial addition to the funds of the Institute.

### A COMPLIMENT TO A HOSPITAL MATRON.

At the annual meeting of the subscribers of the Preston Royal Infirmary, the Chairman of the Board alluded in the warmest terms to the work of the matron, Miss Goffin, and her assistants. He considered that it was owing to her watchful care and to the manner in which she carried out the wishes of the Board in every detail that they met that day in the proud position that the expenses were less than they were last year. In fact, he thought that their matron was second to none in the country. We congratulate Miss Goffin upon the very high compliment paid to her both as the head of the training school and as a woman of business.

### DEATH OF A WELL-KNOWN NURSE.

We regret to hear of the death of Miss Baster, who was for twenty-four years matron of the Royal Berks Hospital, Reading, which took place on Saturday last while she was on a visit at Woodley Hill, Eardley, near Reading. The funeral took place on Wednesday at Bath, where Miss Baster resided since her resignation in 1897. Wreaths were sent by the older members of the nursing staff and by the hospital Board of Management. Miss Baster's death will cause sorrow to many friends and former members of her staff scattered about the country.

### CLAYTON HOSPITAL, WAKEFIELD.

The nurses' examinations in elementary anatomy and physiology and medical and surgical nursing were held at the Clayton Hospital, Wakefield, on Thursday last week, by Mr. I. W. Walker, Honorary Surgeon to the hospital. The results were very encouraging: thirteen probationers entered and all of them passed, special mention being made of Nurses E. B. Fairley, A. P. Gowland, R. Howells, and M. E. Thompson; Nurse Fairley was awarded the examiner's prize.

### LECTURE BY A DISTRICT NURSE.

On Friday evening last, Nurse Harbridge, the Silsden Queen's nurse, gave a lecture in the Town Hall to an appreciative audience of mothers, on "The Care of Young Children." She began by referring to the high rate of infant mortality in England, and explaining how largely this was due to the want of proper care and feeding, arising often, not from wilful neglect, but from ignorance on the part of the mothers. She next gave clear and concise directions as to the management of babies, insisting particularly upon the necessity of absolute cleanliness, both as regards the baby's person and its food and bottles. At the close of the lecture she answered any questions concerning the welfare and care of babies which the mothers liked to ask her. She also sold, to any who wished to buy, several little books and leaflets on the management of children published by the National Health Society.

### The Nursing Outlook.

"From magnanimity, all fears above;
From nobler recompense, above applause,
Which owes to man's short outlook all its charm."

### THE REGISTRATION OF NURSES.

If we are to judge the views of the advocates of nurse registration as recorded in the speeches of the members of the deputation made before the Lord President of the Council, it will be seen that they fail to grapple with the subject in a practical and business-like manner. Mrs. Garrett Fawcett, for instance, insisted that the general public in ever increasing numbers looked forward to the registration of nurses. Why? Because apparently she believes that to set up a register of nurses will ensure adequate protection to the public from all disabilities to which they are liable, owing to the present want of system in the supply and control of private nurses on whom the public must depend. She recorded her personal experience of the conduct of nurses in the course of a three weeks' voyage to South Africa, and maintained that, whilst the conduct of some was excellent, some were shouting for champagne at eleven o'clock in the morning, and to others she would be sorry to entrust the care of a sick dog!

We are bound to say that we are confident that this experience must be practically unique, for in fact the conduct of the overwhelming majority of the nurses in this country testifies that it would be very difficult, if not impossible, to repeat such an experience anywhere on the part of anybody who habitually comes in contact with working nurses up and down the country. Still, accepting Mrs. Garrett Fawcett's view for the moment, what possible remedy can result from the State registration of nurses, for such conduct as she properly condemns, seeing that all the bills and every party for registration recognise and admit that any existing nurse who can produce evidence satisfactory to the central body as regards efficiency and character must, subject to a time limit, be placed upon the register on payment of the registration fee? It was shown conclusively by the evidence before the Select Committee that at least one half, and probably two-thirds, of the nurses available for the public at the present time have not had the opportunity of securing the training or certificates which fulfil the standard likely to be set up by a State board of registration. It follows that if registration were to be enacted by Act of Parliament tomorrow, the public might have even less protection for some years than they possess to-day. To-day any medical man or the head of a household who employs a nurse can insist upon the production of the certificate of training, which enables them to judge of the qualifications and training of any nurse they employ. But the admission as registered nurses of all existing nurses, or the great majority of them, by a central body appointed by the State, would take away the existing protection instead of adding to it, as Mrs. Garrett Fawcett and those who think with her seem to believe.

It follows that a full discussion of the question in all its bearings must convince any Government that before State registration can be set up with safety and advantage to the public and the nurses, steps must be taken, preferably by the co-operation of the nurse-training schools through a voluntary general nursing council, to deal with the majority of working nurses who have not a three years' certificate nor the advantage of having had three years' continuous training. This great body of nurses, many of whom have been trained at the smaller hospitals and elsewhere, is entitled to the fullest consideration, and they should be afforded an opportunity, by presenting themselves for examination through a voluntary nursing council, to enable them to obtain the equivalent of the three years' certificate and to prove their efficiency and capacity as nurses who may be implicitly trusted. In this way, and in this way alone, can full justice be done to the great body of nurses throughout the country, and it is putting the cart before the horse to set up a State register before steps have been taken to winnow and prove the efficiency of every working nurse whose certificates do not fulfil the standard of modern training.

A full discussion should have the further advantage of securing a reconsideration of the present attitude of the authorities of the nurse-training schools who are practically ignored as such in the registration bills which have hitherto been presented to Parliament, and who must in fact, as the education and examining bodies, have direct and adequate representation on any State Board set up for the registration of nurses. Someone must be found with sufficient public spirit to give time to the arduous and absorbing duties which each member of the registration Board will have to devote to the work during the first few years at any rate. The difficulty in this country in regard to this subject has been throughout that the most knowledgeable and influential of the ladies who are at the head of the nurse-training schools have held that they have not the time for public work of this description, owing to the absorbing character of their official duties in connection with the training of nurses at the hospital they serve. Heads of training schools have been supported in this view by the governing authorities, and the result is seen in the unrepresentative character, so far as the actual training of nurses in this country is concerned, of the deputation which waited upon the Lord President of the Council the other day The present position calls for reconsideration and adjustment without further hesitation or delay.

### The Care and Nursing of the Insane.

By Percy J. Baily, M.B., C.M.Edin., Medical Superintendent of Hanwell Asylum.

### I.—ANATOMY AND PHYSIOLOGY.

(*Continued from page 347.*)

As we shall see presently, the left side of the heart has harder work to do than the right side, and therefore the walls of the left side are thicker than those of the right side. For the same reason the walls of the ventricles are thicker than those of the auricles. In association with this we may note the fact that valvular disease of the heart is more common on the left side than on the right side.

In addition to the openings which we have already considered, those, namely, which lead from the auricles into the ventricles, there are others in the walls of these chambers at the origin or termination of blood-vessels which either bring blood to the heart or carry it away from it (fig. 11). In each ventricle

Fig. 11.—Diagram of The Interior of Heart. (Seen from behind).

1. Left Auricle. 2. Left Ventricle.

3. Right Auricle. 4. Right Ventricle.

5. One of the Cusps of the Mitral Valve.

6. One of the Cusps of the Tricuspid Valve.

7. The Openings of the Four Pulmonary Veins.

8. The Opening of the Superior Vena Cava.

9. The Opening of the Inferior Vena Cava.

(7–9: These openings have no valves.)

10. The Origin of the Aorta.

11. The Origin of the Pulmonary Artery.

(10–11: Each of these openings is guarded by three pouch-like valves.)

The arrows show the direction in which the blood flows.

there is one such opening surrounded by three pouch-like membranous valves. Each of these valves is like a little bulging waistcoat pocket, the opening of the pocket looking away from the ventricle into the interior of the blood-vessel. Each pouch is quite close to its neighbour on either side, so that the three form a complete ring round the opening of the vessel which they guard, preventing the return of blood to the ventricle.

The openings in the walls of the auricles differ in number on the two sides; in the left auricle there are four, whereas in the right auricle there are but two. These openings are not provided with any valves.

The blood-vessels are a system of tubes which convey the blood from the heart towards the tissues and from these again back to the heart. Those which carry blood from the heart are called arteries, and those which bring blood back again to the heart are the veins. Between the arteries and veins there is a dense network of minute thin-walled tubes which surround the tissue elements and which are called capillaries. The arteries are continually giving off branches and therefore become smaller and smaller and more numerous the further they are from the heart. They continue to diminish in size until they merge into the capillaries, which are so minute that they require a powerful microscope to see them. Even the prick of a needle is sufficient to wound several of them. The capillaries then gradually unite with one another and so merge by degrees into the minute veins, and these again by uniting with others form the larger veins. Every portion of the body, every muscle, every bone, has its arteries, capillaries, and veins, and even the walls of the blood-vessels themselves are supplied with these vessels.

Now these blood-vessels of ours are not rigid tubes. The walls of the arteries and veins are composed chiefly of involuntarily muscular tissue and what is called elastic tissue; they are consequently elastic tubes, that is to say, if pulled upon they stretch in the direction of the pull and when the pull is relaxed they return to their original shape. The larger arteries have a greater proportion of elastic than muscular tissue in their walls, but the smaller ones, those which immediately precede the capillaries, are almost entirely muscular. By the contraction or relaxation of the muscular fibres the size (diameter) of the vessels is altered, thus allowing a greater or less amount of blood to pass through it as occasion may require.

The structure of the walls of the arteries and veins is almost the same, only those of the veins are very much thinner than those of the arteries. The veins are both less elastic and less muscular than the arteries.

Many veins, especially those of the lower limbs, are provided with valves. These are little pouch-like folds of membrane exactly like those little pockets which surround the origin of the artery which comes off from each ventricle. In the veins, however, the mouth of the pouch looks towards the heart, away from the capillaries. We shall presently see what are the uses of these valves, which form an important difference in the structure of the arteries and veins. In the former vessels they are found only at the point of origin in the heart, whereas in the veins they are found at quite frequent intervals, at all events, in those of the lower limbs.*

Both the arteries and the veins, as well as the cavities of the heart, are lined with a delicate, smooth, very thin, glistening membrane. The walls of the capillaries are composed only of this membrane and they have neither muscular nor elastic tissue, but even they are stretchable so as to allow of a varying quantity of blood to pass through them.

* These valves are not found in the pulmonary veins, the cerebral veins, nor the portal vein.

(*To be continued.*)

### The Nurses' Clinic.

### REST CURES: THE PHYSICAL POINT OF VIEW.

With regard to the actual nursing treatment of rest cases many physicians give definite instructions, but many others leave much to the discretion of the nurse. Assuming the latter to be the case, it will be advisable to consider briefly the following points: (1) Isolation; (2) Rest; (3) Food; (4) Massage; (5) Bathing and Ventilation.

### Isolation.

Practically I have found that almost always those patients do best who, at the beginning of the treatment, are given to understand that complete isolation is part of the cure. It is better, at the very start, to tell a patient that you realise the treatment is a trying one, but that you will do your best to help her through with it, honestly believing that she will find it "worth while," than to let her begin with books and letters, visitors and work, and then gradually take away each of these. The result is usually either depression or rebellion. Of course, there are exceptions, and rigid adherence to a general principle is never to be advocated at any time. In the case of a patient who seems on the verge of being mentally affected, but for whom it is thought that a rest-cure may possibly just save the situation, isolation may prove a great mistake; on the other hand, it may furnish the only possible hope. But this is usually a point for the decision of the physician. In my own work I almost always ask the patient's friends to send her flowers regularly, extracting a promise that no notes shall be enclosed, or simply telling them that the package will be opened by myself. By this means the patient feels that she is in touch with her people, without, as a rule, any undue excitement being caused or a worrying train of thought started.

### Rest.

The question of rest is one that, I think, needs special consideration. We must remember that rest-cure patients are nearly always absolutely tired, in brain and body, often, too, in heart and soul—and we must base the whole of the routine which we propose to carry out on this consideration. We must also bear in mind that what rests one person does not always rest another. For the first two to four weeks our aim should be to induce as much sleep or drowsy restfulness as possible. See that the colours in the room and the general arrangement of it are harmonious, and in tone with the individual patient. Find out, indirectly at first, if anything in the room irritates her, and change it. Do not have the bed near the window during these first weeks, unless the outlook be on to trees and green fields; the patient must be kept lying down, and if excitable will often crave to sit up and look out of the window. Let the light in the room be toned down if there be much nervous excitability or exhaustion; fill the room with sunlight (if possible) at intervals when the patient is of necessity aroused, particularly at meal times; but at other times let the light at first be quiet, and of sufficient softness to prove grateful to the individual disposition, taking particular care that there should never be a strong light or glare in the patient's eyes. Talk to the patient as little as possible at this stage—a few cheery words now and then are quite sufficient—and if there be no special need of watching, let her frequently be alone in the room for half an hour at a time. From the first explain that she must remain absolutely lying down. It is well to allow the patient to get out of bed once a day, at a definite time in the morning, for an action of the bowels, and this opportunity may be taken to make the bed. But this once only in the twenty-four hours, and apart from the weekly weighing there should be absolutely no other exception to the rule of lying down in bed. Try to forestall the simplest wants of the patient, and prevent her from making any exertion whatever. In training young nurses I have often told them to think for the patient as if she were a bad typhoid case, and not allow a single nerve or muscle to do work which they can do for them; yet all this must be done as a matter of course, without any fuss. There must be no sudden sound or sudden movement in the room, otherwise the work of days may be undone in a moment, for many nervous patients will remain for hours in a state of tension after some slight, sudden shake or sound, fearing a repetition. In bad cases it is often better not to allow the patient to feed herself for the first ten days or so. In feeding, if an extra pillow be used, it should comfortably support the whole back and head that no muscle may voluntarily be brought into play. A pillow under the knees will often relieve abdominal tension and induce sleep. In all these matters it is very much better for the nurse to notice what is required and carry it out, than that the patient should have to ask for anything, or make suggestions. She should not be encouraged to discuss her symptoms or sensations, though no symptom, however slight, must escape the nurse's observation. During these first weeks she can often gain a clue to the whole nervous condition of her patient, if she be an accurate, silent, and sympathetic observer of expression, movement, and mood; and this clue should be of the greatest service to her in determining the wisest course of treatment to be pursued in each individual case. No matter how slight the patient's symptoms appear to be, nor whether she only bear a character for fancifulness and indolence, if she is to be treated at all, nurse her thoroughly. The moral effect will be very much better. Let the patient feel that you consider her ill enough to require the treatment, and if your own opinion of her coincides with that of her friends, be rather extra strict as regards the tedium of rest and isolation. But do not treat her as if she were in disgrace; let her feel that at a certain definite time she will get up and be well, and capable of leading a useful, wholesome life. Do not encourage the idea of a convalescent stage with these patients. You want to avoid all idea of invalidism. Tell them now they are ill; but the treatment will make them well, and when they get up they will be quite cured. Keep this idea before them, for facts bear it out, and I have constantly seen a woman who, at the beginning of her cure, could not walk at all, get up toward the end of the sixth week and feel perfectly well, go out for a walk the next day and walk more briskly than the nurse. One warning, however, I must give, and that is that after complete isolation and rest it is most unwise to send the patient straight home to her relations, no matter how well she may seem. If the case has been a severe one, it is best to send her to the seaside or country with a nurse-companion, gradually allowing the relations to join her and the usual life to re-begin. Or in milder cases let her go away with some one—wise friend or relation—until she has got accustomed to the bustle and noise of ordinary life.

### Food.

The feeding of rest cure cases is sometimes very trying both to nurse and patient. I believe that milk and eggs are by far the best forms in which to give nourishment, combined with an average amount of meat, some fish, and

### THE NURSES' CLINIC—*Continued.*

plenty of fruit and vegetables. It is wise to find out at first whether the patient has any special dislikes, and as far as possible avoid them; but if, as is often the case, she begins by saying that she can neither take milk nor eggs, it is useless to promise that these shall not be given. She can be told that as far as possible her tastes will be considered, and, in the case of a patient to whom massage is new, the whole difficulty can often be overcome by assuring her that, whilst being massaged, she will be able to take and digest food, which at other times she certainly could not. A patient can usually be given three pints of milk and two raw eggs per day, besides ordinary meals, from the first day of the treatment, unless her digestion be in an abnormally bad condition. This quantity can be increased daily by ounces until, at the fourth week, she is taking daily from five to nine pints of milk and from four to seven eggs, besides porridge, fish, or bacon, with bread and butter for breakfast; meat, vegetables, and pudding and fruit for lunch; bread and butter, etc., for tea; fish, meat, and vegetables and pudding for dinner. The weekly weight will be a guide as to the necessary amount of food, some patients requiring more than others. Occasionally it is advisable to have a day's rest from over-feeding about the third week. The milk must usually be disguised in various ways, and should partly be given in the form of different foods, such as Benger's, Mellin's, Robinson's groats, cocoa, etc. It should be measured in ounces, and a daily record kept of the exact quantity of nourishment given and the time of each feed. The patient should from the first learn that she must drink the amount of food which the nurse brings to her. If the nurse judge that the patient is really unable to take the full amount due, then she should take less to her bedside for that one time. In all cases it is the nurse who must use her judgment, for if once she allows the patient to refuse food offered, she loses her authority, and endless battles ensue, in which she may be worsted, and which only fatigue the patient. It is better, with a troublesome patient, for a nurse to stand for two hours at the bedside, until the food has been swallowed than to allow the patient to win a battle. But with tact it seldom need come to this, and, if it do, is most rarely repeated if the nurse firmly, but gently, carries her point. At the same time, the nurse must be ever on the watch never to abuse her power. It is possible to be absolutely unyielding in the matter of food, and yet make the patient feel that you sympathise with her discomfort. There is no doubt that the power to take increased nourishment, under conditions of rest and massage, grows, and many patients will soon take over a pint of milk at one feed. The whole quantity of food to be taken at a time should be measured and put into a suitable jug. The jug and a glass should then be carried to the patient's bedside, and only sufficient food poured into the glass at a time for the patient to drink it conveniently. This is a small point, but one worth noting, for I have often seen a full cup or glass given to a resting patient, thus necessitating the use of many nerves and muscles which should have been at rest, to prevent the food from being spilled. In practice, I have found that patients who are not very restless at night get on best if the necessary quantity of food be given in the daytime, so that they have a rest from feeding at night. Patients who are inclined to have suicidal ideas, however, should have food as soon as they awake, in the very early morning, as their worst hours are from about 5 to 11 A.M. Careful attention should be paid to the state of the mouth, and a simple mouth-wash, or even a little plain soda water, may be used after milk feeds.

### Massage.

The amount of massage advisable varies with each case, and a good masseuse should be the best judge in the matter. From ten to twenty minutes is usually safe as a beginning, increased to forty to sixty minutes twice daily. I find it better to rub fairly energetically for forty minutes than to stroke and play at surface movements for sixty minutes. Nervous, irritable patients can stand, indeed will often be soothed by, firm, rhythmical stroking and kneading, but fidgetty, flicking movements should be avoided, and great concentration and care is necessary with these patients, on the part of the masseuse, to ensure a restful, calming result of the daily massage. On the other hand, lethargic patients, who like the sensation of being rubbed and handled, should not be rubbed in too soothing a manner, as the moral effect is not good. For them flicking, rolling, and tapotement is useful, and, as they convalesce they should be encouraged to talk on some subject that will interest them and absorb their attention during the massage. Towards the end of the course of treatment passive movements of the feet, legs, and thighs are useful before the patient walks. In all cases the ordinary sequence adopted in general massage should be followed, but especial attention must be paid to the stomach and abdomen, owing to the abnormal strain on the digestion. Often with these cases there is a long history of constipation, but some of the most apparently chronic of these conditions can be cured during a six-weeks' treatment by careful massage and the use of some simple, non-irritating aperient, such as syrup of figs, which can literally be reduced, minim by minim, until the need for it completely disappears. The necessity for most careful attention to this point cannot be over-estimated, and the permanency of benefit resulting from the cure will greatly depend on the success of this part of the work. The masseuse must carefully note the effect of rubbing, with regard to temperature, pulse-rate, general comfort or malaise, and twitching, and this without the patient's knowledge. It is always best to rub between blankets and to have a hot bottle to the feet while rubbing. In very exhausted cases it may be necessary to have another under the armpits. Very emaciated patients should be rubbed with oil—cocoanut oil is the cleanest in my opinion—otherwise some simple powder may be used. Taylor's Cimolite powder suits almost any skin and is pleasant and does not clog. The masseuse must be careful to stop rubbing before her patient shows signs of exhaustion and should leave her inclined to sleep, after giving some easily taken nourishment and drawing down the blinds. In my opinion it is most unsatisfactory for the same nurse to massage and nurse the case. It is too heavy a strain on one woman, and if the masseuse be a trained nurse she should be able to take responsibility during the nurse's absence. If the patient see no one but the nurse, masseuse, and doctor there should be no undue interruption of the routine.

### Bathing and Ventilation.

A few words on these two important subjects should suffice. It is obvious that if a patient is to remain for six or eight weeks in one room, the air in that room must be changed as frequently as possible. If the patient have sufficient blankets and a hot bottle and a fire in chilly weather, the window need never be closed, and the more fresh air she has about her the better. If unaccustomed to this it can be done gradually, and there is generally little objection, if it be considered as "part of the cure." With regard to bathing, great care must be exercised and patients are better for a thorough wash and sponge between blankets, at least twice a day. Their skin is never in a healthy condition and is often dry and chippy and lifeless. Massage will greatly improve this condition, but thorough washing, with as little movement of the patient as possible must do its part. Particular care should be taken if oil or starch powder be used for massage, to give a hot sponge about an hour afterwards, to ensure that the pores of the skin are not clogged. In some cases of intense nervous irritability a quick cold sponge two or three times a day gives great relief, but the physician should sanction this measure before it is carried out.

In the foregoing remarks it will have been noticed that I have throughout referred to the patient as a woman. I have done this intentionally, for I hold that, generally speaking, it is inadvisable for a woman nurse to attempt to carry out this treatment for a male patient, even though she have the co-operation of a good masseur, and many of the suggestions made above would not be suitable for such a case. To discuss this question is beyond the scope of this paper; but, certainly, if it require an uncommon type of woman to satisfactorily nurse a female patient through a rest-cure, it requires a still rarer type of woman to do the same for a man. There come times, however, when there appears to be no other alternative, and to the nurse who undertakes for the first time this most difficult task I would give one suggestion: approach your task with courage, but, above all, with reverence, and let your own latent motherhood guide you throughout in your treatment of your patient. In his presence every other side of your woman's nature should be as if non-existent. A true mother cannot lose her dignity, yet there is no office that she cannot perform for her sick son, and no warped, distorted word or action of his will be misunderstood by her; able, through her motherhood, to do him any service, she will yet never allow him to lose his self-respect.

As a motto for every rest-cure nurse, I would suggest: "See what your patient really is, then help him, or her, to become it."

### Incidents in a Nurse's Life.

### A "DISTRICT" OPERATION.

Having gone through five years' hospital and infirmary work, I became a probationer once more by entering a training home for the Queen's Institute, to learn the ways and customs of district nursing; in short, how to get the same results without the appliances and comforts of a hospital. I was told off to a very poor district, but not the worst by any means. During my six months I was never without a case at a public house; in fact, I had two at one time—one decent, the other almost a grand one. A public-house was the scene of my first district operation, and I went for three months, Sundays and all, so that it made its impression upon me, and very much amused me too. "The Weavers' Arms" was amidst timber-yards, and was the resort of the workmen (after work) for concerts, which were held in a large room over the bar.

The patient was the landlord, a big, fair man of twenty-nine. His wife and children were very pleasant and agreeable. An old potman was told off to watch for me on Sundays to let me in through the bar. Every day as I passed that bar I was met by respectful salutes from a crowd of workmen, and never once had a rude word or an insolent look.

A senior nurse went with me on the appointed day to show me how it was managed. We were able to make very handy arrangements, the furniture being convenient. The patient was to be put across the bed in the lithotomy position, as the light was very good from the window, the instruments on a mackintosh spread on the dressing table, at the operator's right hand. A stool was placed for him facing the patient, and a mackintosh over the edge of the bed to conduct the fluid into a pail. The lotions, in basins, stood on the marble-top washstand. We hunted up all the dishes, basins, and pails we could lay our hands on, and I made the carbolic lotion for use and a multitude of wool sponges. When all was ready, the senior nurse remembered that she had not thought of the sponges, and seemed quite surprised to think I had made them, unasked. The patient was getting very nervous, his wife was nearly hysterical, and his mother no better. Having procured a supply of hot water, we told off someone to be at hand to bring more water if we called. The doctors now arrived, and the whole thing began.

Having put the patient under chloroform, he was placed across the bed, we nurses standing behind the operator. After a while the assistant (giving the anæsthetic) wanted to see the part to be operated on, and he motioned me to take his place at the patient's head, to continue the chloroform.

"Lift it up every five breaths," he said. "Give one free breath, and give again."

(I remembered that after, when I gave a cat chloroform, to take a bone out of the throat; and it was most successful.)

So there I remained until the operation was over; then I helped to get things straight. We had kept strict silence in the room, and now more water was wanted; so I went to the door and opened it quickly, and three women nearly fell in—the wife, the mother, and the barmaid! They had been listening so eagerly, and so near the door, that they fairly overbalanced, as their support was removed from them.

I could barely help laughing; it was so funny to see them topple over.

At last the doctors left, and we cleaned up. The patient came to, and we left. In the reports in the evening the senior nurse duly reported the operation and the astounding news that Nurse gave the chloroform!

There was a distinct sensation, and many were the remarks of surprise that the doctor should give such a duty to a probationer. When I finished my six months' training the patient was only just convalescent, after a three months' illness; but it was something to feel that my first district operation had had a successful ending.

### To Nurses.

We invite contributions from any of our readers, and shall be glad to pay for "Notes on News from the Nursing World," "Incidents in a Nurse's Life," or for articles describing nursing experiences at home or abroad dealing with any nursing question from an original point of view, according to length. The minimum payment is 5s. Contributions on topical subjects are specially welcome. Notices of appointments, letters, entertainments, presentations, and deaths are not paid for, but we are always glad to receive them. All rejected manuscripts are returned in due course, and all payments for manuscripts used are made as early as possible after the beginning of each quarter.

### Army Nursing in India.

BY A SISTER.

In all the large and many of the small military hospitals there are now sisters. The station where I am at present working is one of the largest, having 270 beds. It consists of eight blocks, each holding 36 beds. These blocks are all quite separate from each other, built in rows about 100 yards apart, one storied, with a verandah running all round, which the patients much enjoy in the cool of the evening, many being carried out in their beds. And the large compound, or garden, which surrounds the whole hospital is one blaze of flowers—chiefly roses from April till November—the wards being made beautiful with fresh flowers every morning. Each block is subdivided; in the centre is the day-room, from which the sisters give out the medicines and stimulants, and the orderlies the milk, and make poultices, etc. On either side of the day-room is a ward of 12 beds—one for the acute and the other the convalescent cases. Beyond these, again, is a small bunk holding four beds, where special cases, or those not expected to recover, are nursed. All these wards open into each other, and also on to the verandah, where inner wire doors, always closed, keep out the flies and mosquitoes. Over each bed is a large square mosquito net. The officers have a block to themselves, composed of little bunks with two or three beds in each. One block is surgical, with a nice little theatre attached; but the work is principally medical—chiefly enteric, dysentery, pneumonia, and malaria. The sisters take charge of all the acute blocks, and the surgical block—usually about five blocks in all—but it varies according to the time of year, the busy season being from May till November. The orderlies trained by the sisters come on in reliefs of four or six every six hours, so arranged that they get six hours on duty and twenty-four off. They usually turn out extremely well, take great interest in their work, are quick to learn, and always patient and kind, which is not as easy as it sounds, when one realises the intense heat that the work has to be done in, the thermometer in the wards often registering 90 and over; while in the plain stations it is often over 105 from June to September, between the hours of 11 A.M. and 7 P.M., and even the nights are not much cooler. But if bad for the workers, how much worse for the poor fever-stricken patients, often with temperatures of 105 and 106, and ice sponging and ice packing go on four-hourly, especially in the enteric block. The sisters live in a bungalow about three minutes' walk from the nearest ward. There is always a senior sister, and from one to six others, according to the size of the station hospital. The junior sisters share the night duty between them, changing every week, as, owing to the heat, flies, and noise of the native servants, sleep in the day time is almost impossible. Every sister is on duty in the morning, each taking a block. There is usually only one afternoon sister, who takes all the temperatures, directs the spongings, etc., and has often a very busy, hot time. The work and life are most interesting, and although at the end of five years I am looking forward to a year's leave in dear old England, still, at the end of that leave, I think I shall be quite ready and willing to return to my work again.

### Wants and Workers.

Will any nurse help another who in her work among poor women and children would be glad of any cast-off underclothing or clothing of any description for them? Address Nurse Martin, 28 Tubbs Road, Harlesden, London, N.W.

### Everybody's Opinion.

[Correspondence on all subjects is invited, but we cannot in any way be responsible for the opinions expressed by our correspondents. No communication can be entertained if the name and address of the correspondent are not given as a guarantee of good faith, but not necessarily for publication. All correspondents should write on one side of the paper only.]

### POVERTY AND TROUBLE AMONGST NURSES.

Miss A. M. Hilder writes from 12 Rose Terrace, Perth, under date of March 20: Thank you for inserting notice about my cloak in your columns. I have already had forty-eight applications, ten of which I received as late as this morning, so I shall probably have more. I have decided on an applicant from this town. It makes one very sad to know of so much poverty and trouble among nurses.

### TRAINED NURSES *v.* UNTRAINED WOMEN.

"A Trained Nurse" writes: Can nothing be done to check the impositions we are daily encountering and which are on the increase? I refer to the untrained women who, with a very slight smattering, probably obtained from nursing lectures, don uniform and go out as fully trained. Personally, it does not affect nurses who are known, but how about our younger colleagues who are both working hard and paying large sums for their training? Surely it would be well if the authorities of our large hospitals could do something to protect the future career of those who are now toiling within the wards?

"Indignant" writes: In last week's issue "A St. Thomas's Nurse" brought the distressing subject of the use—perhaps I should say the mis-use—of out-door uniform before our minds. Her just indignation has been experienced by many of us, but alas! that does not help us. This matter has often been discussed, and very little done to remedy the evil. I do not think that the blame altogether lies on medical men, as "A St. Thomas's Nurse" infers, but rather on the matrons of inferior nursing homes, who employ nurses who may have had a few months in hospital, and proved themselves incapable and unsuitable for hospital work. The majority of nurses, who are considered fit to train in our large training schools, are, I hope, fit to wear uniform without disgracing it. The above remark on nursing homes is not intended in a general sense, nor is any slight intended to private nurses. I merely refer to untrained nurses and the nursing homes that employ them. If a nurse is recommended as capable to the medical man he may omit to inquire what training she has received. Again, I fail to see the "bad form" in wearing out-door uniform. I think it is a great advantage to wear it. It is quickly put on, and not so expensive as ordinary apparel, and should be worn by nurses in training and qualified nurses only. This is where the grievance comes in—the number of persons who wear uniform without any right whatever. Ladies of would-be gentility employ nurse-maids, and wish them to wear uniform, so that they pass as trained nurses. If nurse-maids must wear uniform, why can they not have a distinct style of their own? It would be rather hard lines if all trained nurses had to discard the uniform because untrained persons are allowed to wear it. Can nothing be done for us? I would suggest—if I may be allowed to do so—that all hospitals should adopt the same uniform cloak and bonnet, the same colour, the same style, the same material, these to be chosen by the matrons of our principal training schools. A badge could be worn to distinguish the different hospitals. In proposing that all hospitals should have uniform I do not include either hospitals not recognised as training schools or infectious hospitals. These last should not allow uniform, as it does not save time, the nurses being anyhow obliged to change their dresses. If the above measures were taken any unqualified person wearing our uniform would be liable to punishment and exposure. Civilians do not wear the uniform of the Army or Navy. Why snould our uniform not be accorded the same privilege? I often hear of and see creatures wearing the uniform who are not only a disgrace to nurses, but a disgrace to their sex. Alas! that it should be so.

### INCIDENT IN A MALE NURSE'S LIFE.

"A Male Nurse" writes: Being a reader of your valuable paper and being a male nurse, naturally I take interest in any question on the various cases met with by other nurses. With reference to the article entitled "My Patient," having carefully read all that the male nurse has to say on the subject, and having served several years in a large county asylum and had charge of many suicidal patients, I must say that had that nurse been under me I should most certainly have reported him to the medical officer. When a patient with suicidal tendencies was sent to my ward we signed a parchment not only promising not to let the patient out of our sight, but also to be answerable for his welfare, and a rigid rule was enforced that when any attendant or nurse was in charge of suicidal patients they were on no account to allow the patient to do any work, to use any sharp-cutting instrument, nor to have a handkerchief in bed. The patients, according to this nurse's account, were all jumbled together—throat cases, general paralytic, cancer, and suicidal. Well, I think myself that as the patient seemed so desperately suicidal there should have been a report to the medical officer, and then a nurse put in special charge of the patient, to see that he did not smash the windows and try to get out. I may say that I have seen scores of suicidal patients, but never have I seen a desperately suicidal patient left without a nurse because the nurses were attending to another case.—[We may remind our correspondent that the writer of the article was not describing an asylum conducted on what he considered ideal principles, but an incident exactly as it happened.—Ed. The Hospital.]

### ARE NURSES EXPECTED TO LEARN TOO MUCH?

"Justice" writes: It seems to me a great pity that a whole page of such a valuable paper as yours should be devoted to the grumblings of nurses. I think as the Editor is good enough to give us so much space we might put it to a better account. Just complaints would, of course, be quite a different matter. Lately it has been night nursing; food and off-duty have had their turn. I suppose uniforms and quarters are to follow, as they have not had an airing lately. I do not remember reading any grumbling letters in my pro. days, and I, like all others, did twelve hours night duty, and at one time did seven months at a stretch, and I do not know that I was any the worse for it. I think there are far graver things to be considered. Why do we read almost every week of some nurse committing suicide? Surely something must be very wrong. It has occurred to me that nurses are expected to learn too much. Why should not each nurse qualify in some special department of nursing, and not try to cram in everything? It would be the fairest thing to do, and then more nurses would stand a chance of promotion in their own special direction. To obtain a good appointment now one must train for all branches. A carpenter would not be expected to do bricklayers' and plasterers' work, and yet all are required to build a house. Or, to put it better, would a musician be expected to paint pictures? Yet both are artists. It would take about six years to qualify in all branches of nursing, yet at thirty-five one is considered too old for a good many posts, while at forty it is altogether out of the question. I myself have answered over fifty applications, and have only had an answer to two of them. My testimonials are as good as any nurse could wish to have, and I justly feel that they are no more than I deserve. As I am above the limited age I have at last made myself understand that my nursing career must end. I have been matron in a small hospital, and therefore cannot be taken on as sister or staff nurse. Private nursing I might do for a consideration, say £25, as I am past the age. Of course, experience counts for nothing. Surely this sort of thing needs looking into far more than imaginary woes. I have one comfort—if it may be called so—that is, I am not one by myself. I trust my letter will help to make the present nurses more contented with their lot, and not worry their matrons and everybody else with petty complaints.

### A LONDON NURSING ASSOCIATION IN DIFFICULTIES.

The Chairman and Honorary Secretary of the North London Nursing Association for Providing Trained Nurses for the Sick Poor, 413 Holloway Road, N., writes: May I be allowed to say a few words on behalf of the North London Nursing Association, as the notice in your issue of the 10th inst., although no doubt most kindly meant, is somewhat misleading, and, I fear, may be prejudicial? The present unfortunate financial position, which we feel sure is only temporary, is caused by the large increase of work in 1904-1905. In 1903, 1,402 cases were nursed, whereas in 1904-1905 the cases were 1,642 and 1,582 respectively, the number of nurses on the staff being also larger in these years. The work of nursing the sick poor in their own homes by fully-trained nurses is of such incalculable value, and is so warmly appreciated as a valuable ally of the hospitals, that we feel no effort must be spared to keep our staff of ten nurses up to its full strength. A ball on behalf of the Association is being arranged, most kindly, by Miss Shuttleworth and the Hon. Nina Kay Shuttleworth; and the nurses also are holding a sale of work in July at the home. The responses to many personal appeals which I have made have, so far, this year been most encouraging, but anything which you can do to further our interests will, I need hardly say, be gratefully appreciated. All who know Islington will bear me out when I say that it is becoming poorer year by year. Added to this there is continual increase in the rates, which are becoming a very serious burden. Those facts account for the hard fight which churches and chapels have to finance their various good works, and how difficult it is for them to help adequately such associations as this. At the same time I feel sure the poorer section of the community would welcome an opportunity of giving their mite. Each clergyman and minister has yearly a copy of the annual report and a special letter in which an appeal is made for support. Our annual meeting, which was presided over by the Mayor, supported by Mr. Mower White of the Great Northern Central Hospital, was largely attended and most successful. I apologise for omitting to send you notice of it.

### OVERWORKED PROBATIONERS.

"Fair Play" writes: Reverting to the correspondence in your paper I must crave your indulgence for a few lines in which to reply to "X. Y. Z.," who, in her endeavour to state a case for the hospital authorities and matrons, overreaches herself, and wanders into a wild disquisition on the utter degeneracy, in her opinion, of the present-day nursing profession. It is patent to the most casual observer that "X. Y. Z." is herself a matron, and if the tone of her epistolary effort is an index to her disposition, I think that there is no further need to seek for what she is pleased to call herself, "that tyrant the matron." I would ask her in the first place whether or not the physical condition of the nursing staff does not count for something in "the proper and efficient nursing of the patients?" and if this is of any moment, then is it not the duty of the matron to give some consideration to the conditions under which the physical ability is to be maintained? This would inevitably lead to a proper recognition of the ordinary rules of health and hygiene, and such things as "too long application to routine work," "too little sleep," and "too little outdoor exercise" would become things of the past. Finally, let me ask "X. Y. Z." has she never had a headache, and if so, can she at such times as cheerfully perform her duties? But rather is it not in the interests of even the patients themselves that those who minister to their several necessities should be freed from physical inabilities, however trivial, and thus ensure the brightness which, radiating from the efforts of the nurse, brings with it the gleam of peace and hope to the sufferer?

### NIGHT NURSES' HOURS.

"Gip" writes: In reference to the letter by "A. M. M. B.," I think that the life a nurse lives when on night duty in hospital is bound to be unnatural, for, as "B. M. A. F." says, "every turn is the reverse way of nature." Then, again, the worries and anxieties of night nursing. Although a night nurse may be bright and happy about her work, she nevertheless has a much greater strain on her nerve power than when on day duty, and most people understand what that means. I am at present on night duty (in a small provincial hospital), having fifteen patients in one ward and seven in small side wards to look after. It is my duty to answer the "night bell," and with the night sister attend to out-patients. How often I hurry down the big ward to the bath-room to do some needful cleansing, and wonder if, whilst the water is running, the night bell is ringing. If it does I cannot hear it, though I know that a second ring may waken all in the building, not to mention the anxiety of the waiting visitor outside and perhaps a severe reproof from sister. Do not these little things of necessity worry? Then as to the night meal: how often the latter does more harm than good! A hurried cup of tea or coffee and some indigestible morsel of food. May I not plead for something more nourishing to be allowed to those who watch our suffering fellow-creatures through the night hours? I need not mention the many duties which "M. R." has so admirably set before us, but I quite agree with her statements, although for my part I am perfectly contented with my lot, and, generally speaking, very happy; still I cannot resist pointing out that a night nurse's life is not all that "A. M. M. B." would have some of us believe it to be.

"A SYMPATHISER" writes: I notice that "A. L.," whose letter appears in your columns, does not say whether she considers 12 hours too long for night duty; but I quite agree with her that night nurses' quarters should always be apart from the day nurses' rooms. But at Constance Road Workhouse between two of the night nurses' bedrooms on one corridor there is actually a day nurses' room; whilst the assistant matron's, the cook's, and two other day nurses' bedroms and the "housemaids' cupboard" are all close to their quarters. Consequently someone in discharge of their duty is continually going backwards and forwards to fetch or put things away; the servants are busy cleaning and tidying the various rooms; and there is of course much clatter of pails. For several weeks the workmen have been downstairs; for about the last fortnight they have been on the night nurses' corridor, and though some are as quiet as possible, others are the reverse. Thus for several days the nurses could hardly have got half an hour's consecutive sleep. Of course, work must be done, but those in authority might arrange that all the night nurses sleep either in one block or along a corridor where there are no day workers, and when workmen are about the night nurses should be put for the time being into other rooms.

"X. Y. Z." writes: Will you grant me space for one more letter on the subject of "Overworked Nurses?" We had a hearty laugh yesterday over "Feminine's" description of me, *alias* "X. Y. Z." Her arrow has indeed flown wide of the mark. Of my physical and mental strength I do not propose to write. It is a subject profoundly unimportant to everyone except myself and my committee. But this I will say: I have never been in the habit of going off duty except for serious illness, and, perhaps for that reason, I have not, I confess, very much sympathy for those who do so. My sympathy and my admiration are reserved for those who have the grit to persevere in spite of small ailments. On the other hand, I have had cases of serious illness amongst my staff during the years of my matronship. Two contracted enteric fever during holidays, and went down with it on their return; one contracted diphtheria from a case she nursed here, though she took every precaution. One had a severe attack of lymphangitis, and others have suffered from influenza. I know that not one of these nurses would accuse me of want of sympathy or kindness. They were all cared for to the best and utmost of our ability. But all this is beside the mark. The question is this: "How are the patients to be properly nursed if the nurses themselves go off duty for every small ailment?" This question "Feminine" and her confrères continually evade. They will have to face it if they ever become matrons in their turn. Perhaps they will then solve the problem for us. Until then we must possess our souls in patience. I am glad "Feminine" agrees with me that "prevention is better than cure," only I would start the prevention at an earlier date than she apparently thinks necessary. One thing puzzles me much. Why do these grumbling nurses go in for this work, which no doubt is exacting. No one asks them to do so; no one wants them. Why stay and grumble? We all know the effect that one discontented patient has in a ward, and one discontented nurse is equally demoralising to a hospital staff. If they do stay, for Heaven's sake do let them try to be bright, brave, cheerful workers, a comfort instead of a nuisance to their matron, their sisters, and to the poor patients, who after all, are the *raison d'être* of a hospital.

### NURSES AND THEIR GRIEVANCES.

"H. M. G." writes: I have been sorry to see so much lately in the correspondence *re* nurses and their various grievances, but delighted in your columns of March 10 to read the letter from "Old Westminster." She echoes my sentiments entirely, and I feel sure not only mine, but those of all nurses who have joined the profession, not only for the sake of a living, but from a real love of the work and a desire to help the suffering. I quite admit that night duty is to most a greater strain than day duty, as it is turning the tables of Nature, and she always hates to be upset; but there is so much to compensate one for the extra fatigue, that as a rule all the real nurses that I have met look back to night duty time with pleasure, and I had better explain why for the benefit of those who have not yet discovered the silver lining to their dark cloud. During the daytime (I am speaking of hospital work, not private nursing) there is so much cleaning to be got through, everything done up to time, beds made by a given hour, washings finished, wards dusted, all moving as by clockwork. The patients know this, and seeing so much on hand, looking for sister's first appearance, then for the house doctor's visits, then matron's, and so on all day, their attention is to a great degree taken off from themselves, and other interests fill their minds. Not so in the night. As soon as the lights are lowered there are sure to be some in each ward who begin to be restless and do not try to be quiet; they are anxious to let nurse know that they are awake and likely to have a bad night. Nurse knows these cases well. A few firm words and a cup of hot milk, a pillow shaken up with the cool side topmost, soon soothes these patients, and the result is often a sound sleep all night. Next comes the poor heart case. We know that there will be plenty of work to-night, getting him in and out of bed, and very gently and slowly getting him up, tying him up against a table in a wheel chair, with his arms over a mountain of pillows, whilst gently rubbing the spine; this invariably has a very soothing effect upon these cases, and sleep comes for a time. When this one is settled up for a time there is that poor malignant case, who can sleep all the afternoon amid the day's noise, and then is wide awake at night. Not much can be done until the house physician comes to give the hypodermic injection of morphia; but if all the regular ward work is finished and all else is quiet, just to sit beside this poor sufferer, perhaps heat a piece of flannel and put it over the area of pain; if the legs have become dropsical, to have the time to spare to rub them with methylated spirit and powder to ease the burning—this is a pleasure to the nurse. In the daytime there is so much to be got through that there is no time for these extras. I do not for one moment mean that the patients do not get their due in the daytime; they are just as well looked after, but it stands to reason that when two-thirds as a rule are sleeping, the other third can get the extra little attentions that are so much to a sick person; and it is here that the night nurses have such an advantage over the day ones. Then one more point: Has not every night nurse experienced the look of joy that comes over some face when you go on duty, and the words "Oh, my dear, I've been wanting the night for to come, so as you'd come on duty again," and you know that some poor body really wants you personally? When you come off night duty and form only one of a number entering upon the day's work, you do not get these same sympathetic greetings. I trained some long time ago now, when we lived hard and worked hard, and when grumblers did not get much sympathy, and a happier time in my life I have never had. One grumbler in an institution, setting up the backs of the young untrained members and writing to the Press, instead of going straight to headquarters with her grievances, does an untold amount of harm. Tale bearing is unpardonable; but all just matrons, for their own sake as well as for the good of their particular institution, will, I have always found, be kind and considerate, and listen to any nurse who has a definite grievance to make, and will endeavour to remedy the fault; and I think that as matrons are only human after all, they have even a harder time than either "overworked probationers" or "worn-out night nurses."

### Appointments.

[No charge is made for announcements under this head, and we are always glad to receive and publish appointments. The information, to insure accuracy, should be sent from the nurses themselves, and we cannot undertake to correct official announcements which may happen to be inaccurate. It is essential that in all cases the school of training should be given.]

Acland Nursing Home, Oxford.—Miss Gertrude L. White has been appointed lady superintendent. She was trained at the Bristol Royal Infirmary, and has since been sister and matron of the Memorial Hospital, Almondsbury.

Clayton Hospital, Manchester.—Miss M. Worthington has been appointed staff nurse. She was trained at the Union Infirmary, Willesden.

Gordon Hospital, Vauxhall Bridge Road, London.—Miss Ida Mackintosh has been appointed matron. She was trained at the Sunderland Hospital, and has since been matron of Ascot Cottage Hospital, matron of the Elder Hospital, Glasgow; matron of the Queen's Jubilee Hospital, Fulham Road, London; and matron of Brompton Hospital Sanatorium, Frimley.

Heart Hospital, Soho Square, London.—Miss Alice Stevens has been appointed night sister. She was trained at the General Hospital, Tunbridge Wells, and St. William's Hospital, Rochester. She has since been charge nurse at St. William's Hospital, Rochester; staff nurse at Ruchill Hospital, Glasgow; and nurse matron at the Small-pox Hospital, Rochester.

Huddersfield Infirmary.—Miss Alice Ryley has been appointed sister. She was trained at the Royal Infirmary, Liverpool, and has since been sister of women's surgical ward of the Bury Infirmary.

Lanchester Joint Hospital Board, Tanfield Infectious Hospital.—Miss E. McNaught has been appointed matron. She was trained at the North Western Hospital, Hampstead, and has since been charge nurse at Tanfield Infectious Hospital.

Rotherham Infirmary.—Miss Stansfield has been appointed sister. She was trained at the Bury Infirmary, and has since been staff nurse at the Accident Hospital, Barry Dock; staff nurse at the Royal Chest Hospital, London; and sister of the men's and children's ward of the Bury Infirmary.

Royal Isle of Wight County Hospital, Ryde.—Miss Sadie Hutchinson has been appointed night sister. She was trained at the Hull Sanatorium for Infectious Diseases, and also at the Manchester Royal Infirmary.

### Presentations.

Thompson Memorial Home, Lisburn, Co. Antrim.—Miss E. H. Pringle, lady superintendent of the Thompson Memorial Home, was presented last Friday by patients, nurses, and household staff with a pair of handsome silver entrée dishes, on the completion of twenty-one years as lady superintendent.

### The Nurses' Bookshelf.

A Lecture upon Massage and How to Use a Galvanic Battery in Medicine. By Herbert Tibbits, M.D., Senior Physician at the National Hospital for Paralysis and Epilepsy. (20 pp. Price 1s.)

There is a good deal of useful information in this little pamphlet, for nurses especially in the first lecture and for doctors in the second. There is not, perhaps, much that is new to be said about massage, but the instruction is presented in a condensed form, and may be even learnt by heart, as suggested by the author. "How to Use a Galvanic Battery in Medicine" is reprinted from the *Lancet*, and is only an abstract, but glances briefly at the various manners of applying electricity on patients under many different conditions.

New Charts.

We have received specimens of Harrisson's "Medical Charts" from the Nursing Home, 13 Pembroke Road, Clifton. One is a three-weeks' temperature chart, with space for the usual clinical day; and the other a conveniently spaced table for day and night nursing reports. Both are calculated to prove of practical service. Cases are supplied for the same, so arranged as to hang up either open or closed.

The Nurses' Report Book.

The fifth edition has just been reached of Miss C. M. Löhr's "Nurses' Report Book," which is ruled out for three weeks' day and night work, with temperature charts. Evidently others appreciate this comprehensive and well-arranged book as much as the nurses in Princess Christian's Nursing Home at Windsor, for whom Her Royal Highness ordered two dozen copies. It can be procured from the author, at the Cottage Hospital, Potter's Bar, Middlesex.

### TRAVEL NOTES AND QUERIES.

By our Travel Correspondent.

Convents in Brittany (Nurse C.).—You must tell me how much you can afford to pay per week, and I will give you suitable addresses. I have no recollection of the special one I mentioned, and the Convent terms are very varied.

Accommodation in Cornwall (Burnham).—Send me a stamped and addressed envelope and I will give you addresses. Also tell me if Devonshire would suit you equally well, as I know of suitable quarters there.

Paris in June (Garde-Malade).—June is a very nice month there, but May is still better. You must reckon about £2 5s. for board and lodging per week. Sight-seeing is cheap, but omnibuses are dear; always 30 centimes inside and 15 outside. Electric trams cheap. I think if you want to do all the principal sights you must reckon on £3 per week, but naturally you must regulate sight-seeing by the length of your purse. Hotel Britannique, 20 Avenue Victoria, is good, homely, and cheap. If you are on the third floor pension terms will be 7.50 per day. Another good hotel of the same class is Hotel de Londres et de Milan, 8 Rue St. Hyacinthe. There are several pensions with lower terms, but being further from the centre of Paris, expenses work out at a higher figure in the end.

Rules in Regard to Correspondence for this Section.—All questioners must use a pseudonym for publication, but the communication must also bear the writer's own name and address as well, which will be regarded as confidential. All such communications to be addressed "Travel Correspondent, 28 Southampton Street, Strand." No charge will be made for inserting and answering questions in the inquiry column, and all will be answered in rotation as space permits. If an answer by letter is required, a stamped and addressed envelope must be enclosed, together with 2s. 6d., which fee will be devoted to the objects of "The Hospital" Convalescent Fund. Ten days must be allowed before an answer can be published.

### A Book and its Story.

### SOME IRISH SHORT STORIES.

The short stories in the volume before us vary in interest, but taken as a whole the collection is characteristic of Katherine Tynan's versatility, and proves her able, like every true Irish woman, to make a good story out of slight materials. The "Yellow Domino" has for its hero a gentleman of the Irish guard, Sir Maurice Desmond, in the troubled days when James the Second found a refuge in France through the sympathy of Louis the Fourteenth. He, like his brother officers, had been chosen for height and size. He had fought for James, and when the war was over found himself "the poorest man between the four seas of Ireland, for my house was in ashes, my land seized and sequestrated." In addition he had been separated from the lady of his heart by an obdurate father, who had conveyed his daughter, in company with his money-bags, secretly out of the country before the war broke out, and deposited them at the French Court, where he had a kinswoman in high favour with the Queen. Sir Maurice Desmond has just returned with his comrades from the Low Countries, where he had distinguished himself under Marshal Turenne, when the story opens. The French Queen is giving a masked ball, and thirty Irish officers determine by a ruse to be present. Money was scarce among them, but if enough could be collected for the price of a ticket and a domino, they hoped under this disguise to be able each in turn to be present, and to get some share of the banquet prepared for the guests. "Terence D'Esterre held a hat for the money, and we each cast in what we could, he who had been lucky of late at the gaming table, more; he who had lost, less. We kept no count of the sums, but in the end we had enough to pay for one ticket of admission to the great mask and for a yellow domino. . . . The mask was given for a charity by the Queen's wishes, whence it was called the Queen's Mask, and all of the fashion, of beauty, and of wealth in the capital would pay for admittance, and so swell the coffers of St. Vincent de Paul. . . . We cast lots for order of precedence, and, as it chanced, I came to be last of all. . . . The first cock had crowed before the ticket was thrust in my hand and the yellow domino over my head and shoulders. But the ball was still at its height." Sir Maurice is searching for his lady among the dancers, when he is accosted by a man wearing a cook's dress. He marches him off to the supper room, where at that late hour he scarcely hoped to find such a sight as that which met his hungry eyes. "On every side fountains of wine flowed. The tables blazed with lights, and groaned with venison and boars' heads, and pasties, and all manner of delicate meats, and birds, disguised in some instances beyond the knowing. There was a peacock with his feathers on; there was a roast sucking-pig drowned in wine, and the smell of him very savoury. 'Fall to, most excellent gentleman, fall to!' said the little man. . . . 'How long does the Court stay?' I asked. 'Why, it will dance in the dawn,' he responded."

Sir Maurice, who had been long a stranger to luxury, does full justice to the royal fare, "for the gentlemen of the Irish Regiment more often than not went supperless to bed, since glory was more plentiful in the King's service than Louis d'or, and arrears of pay seemed likely but to grow greater." He is finishing his supper when, turning round, he suddenly becomes aware that the empty banquetting room is filled with a gay throng of ladies and gentlemen, "all very splendidly clad and sparkling with jewels; there was not a mask to be seen. . . . 'Who are you, Master Yellow Domino,' asked he whose hand was on my shoulder, 'that wears the mask after the signal has been given to unmask, and who does such justice to our good things?'" Apparently the news has spread of the huge man behind the yellow domino, who thirty times had sat down to supper. Sir Maurice at once recognises in his interrogator the grande monarque by the stars and orders that he is wearing. At his side stands his lost lady Eleanor, all in white and gold tissue, looking sadder but more lovely than when he parted from her in Ireland. "I gave the soldier's salute. I did not dare uncover. The King gave me another slap on the shoulder. 'Why, you are a most prodigious fellow,' he said. 'Tell us, good Paul, what he has eaten.'" The little cook comes forward, and informs his royal master that since ten o'clock the yellow domino had not been absent for five minutes from the tables, and gave a summary from his tablets of the consumption of wine and food which even for the thirty gentlemen of the Irish Regiment would have been prodigious. The King roared with laughter, but "the Queen and her ladies hid their merriment behind their fans, all except Eleanora, who watched me with a strange intentness, as though she had somehow fathomed my disguise. . . . 'Come,' said the King, 'we must see your face; off with the domino! You are one of the wonders of the world.' . . . 'Sire,' I said, 'before I uncover may I explain to your most excellent majesty how it came that I ate as for thirty?'" He asks clemency of his majesty for himself and comrades. "Oh, oh!" said the King, looking a little disappointed. "So you have not eaten all the food yourself then?" "Sire," I replied, "no man could do it and live." The King then demands his name and who are his comrades. Sir Maurice replies by giving his name and rank as captain in his majesty's Regiment of Horse, adding, "the yellow domino is not only myself, but twenty-nine other gentlemen of the regiment." . . . "Are there any more of you to sup?" he asked, his lips twitching. "I am the last of the thirty, sire," I said. "Why, heaven be praised for that," he responded, "or else we should have a famine in our kitchen." The King becomes more serious and glances round, makes the inquiry if anyone present knows Sir Maurice Dering. "There was a little movement in the crowd, but before anyone could speak my Eleanora stepped forward. 'Sire,' she said, 'Sir Maurice Desmond is a most brave and honourable gentleman, who lost his all fighting for King James in Ireland.' Her voice trembles a little, but she goes on frightened as a fawn, yet brave as a martyr. 'We were dear friends once . . . but the fortunes of war separated us. I have never ceased to look for my friend . . . yet never thought to find him in your Majesty's Irish Regiment.' 'I think, sir,' said the King, 'if I were you I would unmask and kiss the lady's hand; she has been cold to all the world while she waited for her friend.'"

Of the other stories one of the best is an essentially Celtic one, called "The Heart of the Hill," a romantic setting to a romantic story of love at first sight between a man and a girl, who, though near neighbours—their property adjoins—until fate and an hour of peril combine to throw them together, had cause to regard each other (over some dispute as to rival claims to a portion of land) as enemies—at least, from the girl's point of view. Her unknown preserver is the man whose name had become hateful to her. But she loves him before she finds out this, and a very pretty romance ensues.

* "The Yellow Domino." By Katherine Tynan. (F. V. White and Co. 6s.)

### Notes and Queries.

**REGULATIONS.**

**The Editor is always willing to answer in this column, without any fee, all reasonable questions, as soon as possible.**

**But the following rules must be carefully observed.**

**1. Every communication must be accompanied by the name and address of the writer.**

**2. The question must always bear upon nursing, directly or indirectly.**

**If an answer is required by letter a fee of half-a-crown must be enclosed with the note containing the inquiry.**

*Missions.*

(195) Can you give me full particulars of the Nurses' Missionary Union? Also, are any callings for nurses (trained or otherwise, especially those holding the L.O.S. certificate) in foreign mission fields, or in what way might one combine the two callings?—*W. S.*

Perhaps Miss Dashwood, 5 Cambridge Gate, Regent's Park, N.W., can help you to the address you require. With regard to your second question, apply to any of the great Missionary Societies, to the Missionary Training Home for Ladies, 10 College Terrace, Brighton; or consult a copy of Burdett's "Hospitals and Charities," or "The Englishwoman's Year Book." The Scientific Press, 28 Southampton Street, Strand, will procure them for you.

*Queen's Nurses.*

(196) Will you kindly let me know whether any parish would be compelled to appoint one of the Queen's Nurses as a district nurse, because the parish receives the sum of £10 annually towards the nurse's salary? I am a trained nurse, possessing a three years' certificate, and would stand a fairly good chance of getting the post as my home is in the neighbourhood, but I do not belong to the Queen's Nursing Institute. If that is essential, would you also let me know what time I would have to serve as a Queen's nurse before I could take on district nursing?—*J. H.*

The parish would, we think, certainly prefer to engage a Queen's nurse. If you hold a certificate from an approved general hospital, it will not be difficult for you to join the Institute. Write for full particulars to the General Superintendent, Queen Victoria's Jubilee Institute for Nurses, 120 Victoria Street, S.W.

*Maternity Nurse's Fee.*

(197) A lady engaged me to nurse her from January 26th but when near the time the doctor for the case wished me to go in on the 14th. I did so, but had to give up two weeks' work in consequence. The baby did not arrive till February 6th. Now the patient only wishes to pay me from the time she engaged me—January 26th. Can I claim for the other 12 days?—*Nurse J.*

Certainly you can do so if you have any proof that the doctor engaged you at the lady's desire. If nurses would be careful to make their arrangements with their patients in writing these frequent troubles would be avoided.

*Convalescent Home for Nurse.*

(198) I am just recovering from a sharp attack of pneumonia. Can you tell me of a free Convalescent Home? I am a nurse, but being 55 years of age have not been so regularly in work of late, so have spent most of my savings.—*Nurse T.*

Perhaps the Matron, Parkwood, Henley-on-Thames, may be able to help you, or The Merchant Taylors Convalescent Home, Hothamton Place, Bognor; Thomas Bantings Memorial Home, Parade Lodge, Marine Parade, Worthing; or H. Partridge, Esq., Castle Hill, Bletchingley, Surrey.

*Asylum Patient.*

(199) Can you give information of a Convalescent Home, near Liverpool or London, suitable for a young lady of limited means, for brief period, on her discharge from a private asylum, preparatory to a situation.—*I. H.*

Write to the After-Care Association for Persons Discharged from Asylums, Church House, Dean's Yard, Westminster, S.W.

**Handbooks for Nurses.**

Post Free.

"How to Become a Nurse: How and Where to Train." 2s. 4d.

"Nursing: its Theory and Practice." (Lewis.) ... 3s. 6d.

"Nurses' Pronouncing Dictionary of Medical Terms." 2s. 6d.

"Complete Handbook of Midwifery." (Watson.) ... 6s. 4d.

"Preparation for Operation in Private Houses." ... 0s. 6d.

Of all booksellers or of The Scientific Press, Limited, 28 & 29 Southampton Street, Strand, London, W.C.

### For Reading to the Sick.

"OPEN OUR EYES."

Open our eyes, Thou Sun of life and gladness,
That we may see that glorious world of Thine:
It shines for us in vain while drooping sadness
Enfolds us here like mist; come, Power benign,
Touch our chilled hearts with vernal smile,
Our wintry course do Thou beguile,
Nor by the wayside ruins let us mourn,
Who have the eternal towers for our appointed bourne.

*John Keble.*

Let us serve God in the sunshine, while He makes the sun shine. We shall then serve Him all the better in the dark, when He sends the darkness. It is sure to come. Only let our light be God's light, and our darkness God's darkness, and we shall be safe at home when the great nightfall comes.—*F. W. Faber.*

Why should we be anxious for a long life, or wealth, or credit, or comfort, who know that the next world will be everything which our hearts can wish, and that not in appearance only, but truly and everlastingly? Heaven is at present out of sight, but in due time, as snow melts and discovers what it lays upon, so will this visible creation fade away before those greater splendours which are behind it, and on which at present it depends. In that day shadows will retire, and the substance show itself. These are thoughts to lead us to rejoice in every day and hour that passes, as bringing us nearer the time of His appearing, and the end of waiting, darkness, disputing, sorrow, and care.—*J. H. Newman.*

It is God's will that we should do whatever lies in our power to attain all holiness; but we must remember that though we may plant and water, God only can give the increase, and therefore we must leave the fruit of our efforts to His good Providence. So if we do not make such conscious progress in the spiritual life as we should desire, there is no need to be disturbed and anxious; better far it is to be calm, doing diligently all that depends upon ourselves, but leaving results to our dear Lord. The labourer will be called to account for his careful cultivation, not for the abundance of his harvest. Meanwhile, do the work which concerns you at the present moment; go on quietly with your spiritual exercises, give yourself up many times each day, both heart and mind, into God's Hands, commending your work humbly to Him. Consider what daily opportunities you have of serving Him, whether by your own advance in holiness, or by promoting that of your neighbour, and make a faithful use of all such.

*S. Francis de Sales.*

What then? I am not careful to inquire:
I know there must be tears, and fears, and sorrow,
And then a loving Saviour drawing nigher,
And saying, "I will answer for the morrow."
What then? for all my sins, His pardoning grace;
For all my wants and woes, His loving-kindness;
For darkest hours, the shining of God's Face,
And Christ's own Hand to lead me in my blindness.

*Clewer Manual.*

## Figures and Tables

**Fig. 11. f1:**